# Central New York Homœopathic Society

**Published:** 1882-06

**Authors:** C. P. Jennings

**Affiliations:** Secretary


### CENTRAL NEW YORK HOMŒOPATHIC MEDICAL SOCIETY.

MEETING HELD AT SYRACUSE, N. Y., MARCH, 1882.

The stated quarterly meeting of the society was held at the office of Dr. W. A. Hawley; Dr. S. Seward, President, occupied the chair. Present: Drs. Seward, Boyce, Brewster, Jennings, Bigelow, Gwynn, Ewens, L. B. Wells, Nash, Young and Besemer.

After the minutes of the last meeting were read and approved, Drs. Brewster and Boyce were appointed as committee on credentials, and they then recommended for membership Drs. Charles L. Swift and Daniel W. Clausen, who were elected. A paper by Dr. E. P. Hussey was then read, and the thanks of the society were voted him for the model character of his report, and the Secretary was directed to publish the paper, with the approval of the society. Dr. Brewster then read a clinical paper, which was accepted, with the thanks of the society and ordered to be published.* Dr. Seward

---

* The papers of Drs. Hussey and Brewster are published in this issue under CLINICAL BUREAU.—Editor H. P.

expressed himself as grateful for having reports of cases treated with high potencies made so clear.

Dr. Boyce was glad to hear such papers read, regardless of the potency used. They go to show the effectiveness of genuine Homœopathy. Dr. Boyce then read a letter from Dr. P. P. Wells, in which he stated that he had been informed that this society had dropped the name of homœopathic. The Secretary was instructed to inform Dr. Wells that the society still retains the name and maintains the principles of genuine Homœopathy and that the thanks of the society are tendered him for his kind offer of a paper for the June meeting. Paragraph 206 of the *Organon* was then taken up for discussion.

Dr. Boyce said that Hahnemann maintained that the most difficult cases were those which had been complicated by previous medication.

Dr. Seward thought that few took the pains that Hahnemann took.

Dr. Nash wished to know what rule decides the anti-psoric character of a remedy. Would Hahnemann consider only anti-psoric remedies if he saw a case was psoric; should not the remedy correspond with the peculiar symptoms of the case?

Dr. Seward: Hahnemann says there must be correspondence of symptoms.

Dr. Boyce: My supposition is that Hahnemann found certain remedies produced alleviation only, while cures came from what he called anti-psoric.

Dr. Nash: He speaks somewhere of cases where the seemingly indicated remedy did not cure and he found cure follow the administration of anti-psorics.

Dr. Gwynn thought the anti-psoric may bring the system into a better condition for the action of the remedy. For example, Sulphur.

Dr. Boyce: Hahnemann speaks of other remedies than Sulphur. There must be some reason for giving Sulphur. No remedy is to be given without proper indications.

Dr. Gwynn: Sulphur is apt to cover most cases of a psoric taint and will prepare the system for some other remedy.

Dr. Boyce: Of paragraph 212, I wish to say we are given the modalities. There is a character to every remedy. Mental characteristics are distinct and marked. Belladonna is often written on the face of a patient; so is Aconite or Pulsatilla. When the mental condition is prominent we hardly need go a step farther, no matter what the pathological condition. The mental condition is a wonderfully accurate guide to the remedy.

Dr. Nash: Some diseases do not produce prominent mental symptoms and there are remedies of the same character. I was called to a man having great pain in the ileo-cœcal region, with retching when getting up; could not lie still; face red while lying, very pale on rising. Aconite, in water, repeated every fifteen minutes, relieved him in two hours.

Dr. Boyce: Why repeat?

Dr. Seward: Give to get a quick effect.

Dr. Gwynn: Hahnemann, in his *Chronic Diseases*, advises repetition. As to mental conditions, they are important. As to our dates, here is a difficulty, where there is anything in nature that leads to attenuation. I do not deny the efficacy; but what is the reason for potentiation?

Dr. Bigelow: Miasms are attenuated.

Dr. Boyce: Experience only has led to dynamization. Hahnemann found inert substances developed energy by attenuation. Some say that attenuations produce no effect on the healthy; but the Austrian proving of Nat. mur. 30, produced more symptoms on the healthy than lower preparations.

Clinical papers from Drs. S. Swan and C. Lippe were then read and the thanks of the society tendered those gentlemen.*

Dr. L. B. Wells: Some of our best physicians are in the habit of repeating the dose. I knew a patient of the late Dr. Dunham, who was directed to take a dose of the remedy given twice a day. He gave to me in a chronic trouble a dose thrice daily for three or four weeks.

Dr. Boyce: I noticed in a medical journal, a short time ago, that almost all the cases were treated by the repetition of the dose. The question is, when to repeat?

Dr. Wells: The first dose sets up curative action and demolishes the disease. The doses afterward given are of no consequence; they do no damage to healthy organs, unless there be peculiar susceptibility.

---

* The papers of Drs. Lippe and Swan were published in April number of this journal.—Editor H. P.

Dr. Seward: There must be foundation in fact for that, or we would lose many patients.

Dr. Boyce: I gave Sepia$^{2c}$, three doses in an afternoon, to a patient; to another a fourth dose. Pathogenetic effects showed themselves unmistakably.

Dr. Nash then read the symptoms of Aconite from Hoyne's cards. "Fear of death" has led Dr. Besemer to prescribe Aconite, and with success. Dr. Nash had often removed excessive fear of death with Aconite; also fear of going into a crowd; anxiety in the region of the heart is another marked symptom of Aconite.

Dr. Boyce: The difference between Aconite and Lachesis is that the Lachesis patient dies when he says he will.

Dr. Nash: I have cured that symptom in typhoid fever with Lachesis. Aconite cures when a child is angry.

Dr. Besemer: I would use Chamomilla for irritable, long-lasting anger; Aconite for quick temper.

Dr. Nash: Loss of memory suggests Anacardium; Sulphur, cannot remember names; Aconite, cannot remember dates.

Dr. Boyce: As to fear of rising up, the difference between Aconite and Bryonia is that Aconite has a red face, Bryonia is nauseated and faint; both have vertigo on raising up; Cocculus also has it.

Dr. Swift tells me that Aconite has vertigo on entering a warm room; Lycop. in a hot room.

Dr. Wells: Fullness in forehead, as if the brain would push out, is Aconite; Bryonia has it on stooping and bending over.

Dr. Boyce: Aconite should not be given in febrile conditions where patient is quiet.

Dr. Wells: Hahnemann's way of preparing Aconite was the expressed juice of the stems and leaves, at flowering time, mixed with equal parts of alcohol. Several cases of recovery from collapsed conditions were reported as brought about by Aconite. Aconite is effective in the first stages of inflammatory dysentery.

It was then ordered that Aconite and Actea be studied at the next meeting, and that Bœnninghausen's article on Hahnemann's *Three Precautionary Rules* be taken up instead of the *Organon*. The society then adjourned.

[The case given by Dr. Nash at the last meeting, of apoplexy cured by Conium, should read as having been cured by Dr. Ad. Lippe.]

C. P. JENNINGS, *Secretary*.

